# SOX6 suppresses the development of lung adenocarcinoma by regulating expression of p53, p21^CIPI^, cyclin D1 and β‐catenin

**DOI:** 10.1002/2211-5463.12762

**Published:** 2019-12-12

**Authors:** Liyan Lv, Min Zhou, Jian Zhang, Fang Liu, Li Qi, Shuai Zhang, Yi Bi, Yan Yu

**Affiliations:** ^1^ Department of Oncology The First Affiliated Hospital of Harbin Medical University China; ^2^ Department of Pathology The First Affiliated Hospital of Harbin Medical University China; ^3^ Department of Thoracic Surgery The First Affiliated Hospital of Harbin Medical University China; ^4^ The Sixth Department of Medical Oncology Harbin Medical University Cancer Hospital China; ^5^ Department of Radiation Oncology The Second Affiliated Hospital of Harbin Medical University China; ^6^ Hemodialysis Department Heilongjiang Provincial Electric Power Hospital Harbin China

**Keywords:** β‐catenin, cyclin D1, lung adenocarcinoma, p21^CIPI^, p53, SOX6

## Abstract

The Sry‐related high‐mobility group box6 (SOX6) has been implicated in the development of cancer, but its role in lung cancer is incompletely understood. Here, we report that SOX6 expression is frequently down‐regulated in lung adenocarcinoma tissues. Moreover, SOX6 can inhibit the proliferation and invasion of lung adenocarcinoma cells, which may occur through cell cycle arrest at G1/S due to up‐regulation of p53 and p21^CIPI^ and down‐regulation of cyclin D1 and β‐catenin. Univariate and multivariate analyses revealed that the expression of SOX6 is significantly associated with patient disease‐related survival and is an independent prognostic factor for lung adenocarcinoma. These data suggest that SOX6 may act as a suppressor of lung adenocarcinoma.

## Graphical abstract

We found that Sry‐related high‐mobility group box6 (SOX6) may be a tumor suppressor in lung adenocarcinoma, and prognosis of patients with lung adenocarcinoma with low SOX6 expression is poor. In this study, SOX6 inhibits the G1/S phase transition of cell cycle by up‐regulation of p53 and p21^CIPI^ and down‐regulation of cyclin D1 and β‐catenin expression, thereby inhibiting the proliferation, migration and invasion of lung adenocarcinoma cells.

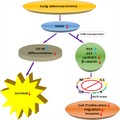

**Abbreviations**
MTT: 3‐(4,5‐dimethyl‐2‐thiazolyl)‐2,5‐diphenyl‐2‐H‐tetrazolium bromide
NSCLC: non‐small‐cell lung cancer
SOX6: Sry‐related high‐mobility group box6

Lung cancer is the main cause of cancer death worldwide, and its incidence is the second highest among all cancers 1. Non‐small‐cell lung cancer (NSCLC) represents approximately 80% of lung cancers. At present, the pathogenesis of lung cancer is not completely clear, and the prognosis of lung cancer is still poor because of its high degree of malignancy, rapid progression and the frequent detection of distant metastasis at initial diagnosis 2, 3. Therefore, further exploration and discovery of new target genes of therapeutic value is of great significance for improving the diagnosis and prognosis of lung cancer.

The Y‐related transcription factor family (SOX) includes 30 members, and Sry‐related high‐mobility group box6 (SOX6) belongs to the D group with SOX5 and SOX13 4, 5. SOX6 is specifically expressed during the development of the central nervous system and the formation of early embryonic cartilage and muscle tissue, and plays an important role in cell growth, development and proliferation 6, 7. Recent studies have also indicated that the SOX6 acts as an oncogene or tumor suppressor in various cancers, and expression of SOX6 is closely related to the occurrence, development and prognosis of human tumors 8, 9. Schlierf *et al*. 10 and Ueda *et al*. 11 found that during the early stage of gliomagenesis, SOX6 acts as an oncoprotein that induces the enhancement of transcriptional regulation, leading to high levels of SOX6 in the tissues and sera of patients with glioma. Other discoveries have shown that SOX6 acts as a tumor suppressor in prostate cancer, esophageal squamous cell carcinoma, hepatocellular carcinoma and pancreatic cancer 12, 13, 14, 15. Furthermore, the down‐regulation of SOX6 expression is closely related to the poor prognoses of hepatocellular carcinoma and esophageal squamous cell carcinoma 8, 9.

Many studies have investigated the role of SOX5 in lung cancer. A recent study showed that SOX5 is required for the development of the lung, and that SOX5 can drive the malignant phenotype of NSCLC cells 16. SOX5 can promote the metastasis of lung adenocarcinoma through the epithelial–mesenchymal transition and is an adverse prognostic factor of lung adenocarcinoma 17. The amino acid sequences of the high‐mobility group boxes of SOX5 and SOX6 are highly similar, suggesting that SOX5 and SOX6 might perform similar functions in certain cells 18. In contrast with the research in SOX5, studies on the role of SOX6 in lung cancer are relatively rare. One study identified SOX6 as a target of miR‐1269a in NSCLC, and miR‐1269a promotes NSCLC growth by down‐regulating the expression of SOX6 19. The precise role of SOX6 in lung cancer and its related molecular mechanisms remain to be identified. Here, we investigated the expression of SOX6 in lung adenocarcinoma and its role and potential mechanism of action in tumor development.

## Materials and methods

The experimental protocol was approved by the Institute of Laboratory of Hematology and Cancer Center of The First Affiliated Hospital of Harbin Medical University. All methods were performed in accordance with the approved guidelines, and written informed consent was obtained from all patients. All procedures performed in studies involving human participants were in accordance with the ethical standards of the institution, the National Research Committee, or both, and with the 1964 Declaration of Helsinki and its later amendments or comparable ethical standards.

### Patients and tissue specimens

Thirty pairs of lung adenocarcinoma tissues and adjacent nontumor tissues were acquired from the Department of Thoracic Surgery of The First Affiliated Hospital of Harbin Medical University and the Department of Thoracic Surgery of Heilongjiang Cancer Hospital. Thirty pairs of fresh tissue were removed surgically and frozen in liquid nitrogen immediately after surgery. The tissue samples were stored in a refrigerator at −80 °C. Paraffin‐embedded tissues (*n* = 145) were obtained from patients who underwent surgery for lung adenocarcinoma at the Department of Thoracic Surgery of The First Affiliated Hospital of Harbin Medical University from January 2009 to January 2012. The patients did not receive treatment before surgery. Patients granted their informed consent to participate in the study. Clinical data of patients included in this study are detailed in Tables S1 and S2.

### Immunohistochemistry

Immunohistochemistry was performed according to published protocols. Tissue sections were deparaffinized, blocked with 10% serum for 10 min and then incubated with SOX6 Ig (1 : 500, ab30455; Abcam, Cambridge, UK) at 37 °C for 30 min and then overnight at 4 °C. Sections were then incubated with secondary Ig (PV9000; ZSGB‐BIO Inc., Beijing, China). The levels of SOX6 in the nuclei of normal lung and lung adenocarcinoma cells were evaluated semiquantitatively according to the previously published protocols. Sections were evaluated and scored for the degree of SOX6‐positive staining as follows: 0, no staining; 1, light staining (light yellow); 2, moderate staining (yellow); or 3, strong staining (yellow‐brown). Sections were evaluated for the percentage of stained cells as follows: 0, <5%; 1, 5%–25%; 2, 25%–50%; or 3, >50%. The staining degree score was multiplied by the score for the percentage of stained cells. Sections with overall scores of 0–3 were negative, and sections with overall scores ≥4 were positive. The stained tissue sections were evaluated using light microscopy (original magnification ×200 or ×400). According to the pathological diagnostic criteria, the degrees of differentiation of lung adenocarcinoma are defined as follows: well differentiated (>95% gland duct formation), moderately differentiated (50–95% gland duct formation) and poorly differentiated (0–49% gland duct formation). Two pathologists who were uninformed of the patients’ clinical data assessed the data for the nuclear expression of SOX6.

### Real‐time quantitative RT‐PCR assay scores

Total RNA was extracted using TRIzol reagent (TIANGEN Biochemical Technology Co., Ltd, Beijing, China) according to the manufacturer’s instructions. The concentration and purity of the RNA preparations were determined using a NanoDrop ND‐2000 spectrophotometer (Thermo Scientific, Shanghai, China) and denaturing agarose gel electrophoresis. The PrimeScript cDNA RT Reagent Kit with gDNA (Eraser RR047B; Takara Bio Inc., Dalian, China) was used for reverse transcription, and the reactions were performed according to a published protocol. The PCR primer sequences (Invitrogen, Carlsbad, CA, USA) were as follows: SOX6 sense and antisense primers (GenBank: AF309034.1), (forward) 5′‐GGGAAAGTCAAATGAAGATGGAA‐3′ and (sox6 reverse) 5′‐CGCTTAATGTGTGGCTCGCT‐3′; 18S ribosomal RNA sense and antisense primers (GenBank: MG595728.1) (forward) 5′‐GTAACCCGTTGAACCCCATT‐3′ and (reverse) 5′‐CCATCCAATCGGTAGTAGCG‐3′.

### Western blotting

Radioimmunoprecipitation assay (R0278; Sigma‐Aldrich, St Louis, MO, USA) protein extraction reagent was used to extract total cellular and tissue proteins, and the bicinchoninic acid method was used to quantitate the amount of protein. Detection of immune complexes was performed according to a published protocol 20. Antibodies were diluted as follows: anti‐SOX6 (ab30455; Abcam), anti‐p53 (1C12; 2524; 1 : 500; Cell Signaling Technology, Danvers, MA, USA); anti‐p21^CIPI^ (60214‐1‐1g, 1 : 1000; Proteintech Group Inc., Wuhan, Hubei, China), anti‐catenin (66379‐1‐1g, 1 : 1000; Proteintech Group Inc.) and anti‐cyclin D1 (60186‐1‐1g, 1 : 1000; Proteintech Group Inc.).

### Cell culture and transfection

The lung cancer cell lines A549 and HCC827 were obtained from the Laboratory of Hematology and Tumor Center of The First Affiliated Hospital of Harbin Medical University and approved by the Laboratory of Hematology and Tumor Center of this hospital. All methods were performed in accordance with the approved guidelines. A549 and HCC827 cells were cultured at 37 °C in 90% RPMI 1640 culture medium (Invitrogen) containing 10% FBS and 1% penicillin–streptomycin in an atmosphere containing 5% CO_2_.

To establish stable cell lines, we transfected cells with the PCMV6‐Entry SOX6 expression vector (RC227487; OriGene Technologies Inc., Rockville, MD, USA) using Lipofectamine 2000 Transfection Reagent (Invitrogen) according to the manufacturer’s instructions. Cells (1.5 × 10^5^ cells·mL^−1^) were plated the day before transfection and then cultured for at least 12 h before transfection. Clones that stably expressed SOX6 (SOX6‐A549 or SOX6‐HCC827) were pooled, and the SOX6 cDNA prepared from each pool was resequenced. A549 and HCC827 cells transfected with the empty vector (Vec‐A549 and Vec‐HCC827) were used as controls.

### Flow cytometry

Cells were resuspended in serum‐free medium and adjusted to 1 × 10^4^ cells·mL^−1^; the cells were centrifuged and washed. The cell density was then adjusted to 1 × 10^6^·mL^−1^ with 10 mm PBS, and 100 µL of the cell suspension was aliquoted into a separate tube. Next, 400 µL of cell cycle detection reagent (558662; BD Biosciences‐CN, Shanghai, China) was added, and the reaction was incubated at room temperature for 30 min. The cells were centrifuged, resuspended in 500 µL of 10 mm PBS, and analyzed using flow cytometry (BD FACSCanto II, Franklin Lake, NJ, USA). The cell cycle was analyzed using flowjo software (Leonard Herzenberg laboratory of Stanford University, Stanford, CA, USA).

### Cell proliferation assay

Cell proliferation was determined using the 3‐(4,5‐dimethyl‐2‐thiazolyl)‐2,5‐diphenyl‐2‐H‐tetrazolium bromide (MTT) method. Cells were added to a 96‐well cell culture plate (100 µL per well). The MTT assay was performed after 48 h, and the transfection medium was replaced according to the manufacturer’s instructions. Attenuances were measured at *D*_570 nm_.

### Colony formation assay

Cells transfected for 24 h were trypsinized, and the cell density was adjusted to 1 × 10^4^ cells·mL^−1^. The suspension was added to six‐well cell culture plates (20 µL per well), and colonies were counted after 14 days (>50 cells per colony).

### Wound healing assay

Before the transfected cells changed their fluids, we used a yellow micropipette tip to introduce a ‘wound gap’ into the cell layer in the wells of a six‐well cell culture plate. The cells were washed and growth medium was added. The cells were photographed (original magnification ×100) at 0, 24 and 48 h.

### Transwell migration assays

Transwell polycarbonate membrane cell culture plate inserts (8 µm; CLS3422; Sigma‐Aldrich) were used for the cell migration assay. Twenty‐four hours after transfection, the cell density was adjusted to 5 × 10^6^·mL^−1^. Culture medium (500 µL) was added to the lower chamber of the Transwell insert, and 50 µL of Matrigel was added to the upper chamber. After 24 h, the cells were fixed with paraformaldehyde and stained with crystal violet. Photographs were taken of the migrated cells, and the numbers of cells in 200 fields were counted.

### Statistical analysis

Statistical analysis was performed using spss 19.0 software (SPSS Inc., Chicago, IL, USA). The relationships between the expression of SOX6 and patient clinicopathological features were evaluated using the chi‐square test. The Student’s *t*‐test was performed to evaluate significant differences between control and experimental groups. The Kaplan–Meier method was used to analyze survival, and the data were compared with those acquired using log analysis. The relationships between SOX6 expression and clinicopathological parameters, and the prognosis of patients with lung adenocarcinoma were assessed using univariate and multivariate analyses 21, 22, 23. A *P*‐value <0.05 indicated a significant difference.

## Results

**Figure 1:**
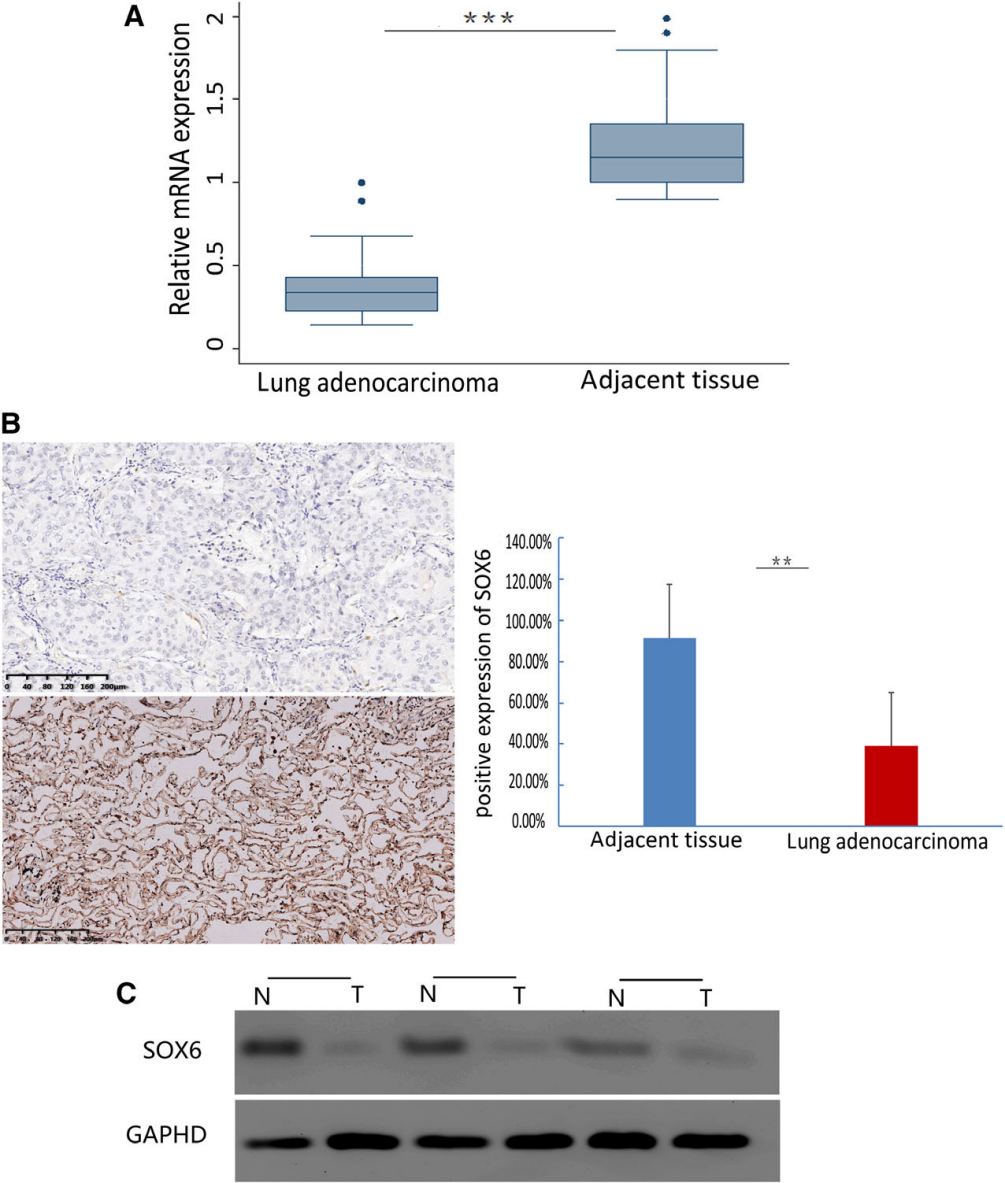
RT‐PCR, immunohistochemical, and Western blot analysis of SOX6 mRNA and protein expression in a lung adenocarcinoma. (A) Expression of SOX6 mRNA was significantly down‐regulated in 30 pairs of lung adenocarcinoma tissues compared with their respective adjacent nontumor tissues. ***P < 0.001. (B) Representative data showing immunohistochemical detection of SOX6 expression in a pair of lung adenocarcinomas (top left) and adjacent nontumor tissues (bottom left; brown in the nucleus). Percentage of SOX6‐positive cells in lung adenocarcinoma tissues versus their respective adjacent nontumor tissues (right). The Student’s *t*‐test was performed to evaluate significant differences between control and experimental groups. Error bars represent SD. **P < 0.05. Scale bars: 200 μm. The sections were counterstained with hematoxylin (original magnification ×200). (C) Western blot analysis showed that the expression of Sox6 in lung adenocarcinoma was down‐regulated compared with that in adjacent tissues.

We used RT‐PCR to detect the expression of SOX6 mRNA in lung cancer samples. We examined SOX6 mRNA in 30 pairs of adjacent nontumor tissues and lung adenocarcinomas, and detected significantly higher levels in the nontumor tissues (22/30, 73%); the difference in SOX6 mRNA levels was statistically significant (*P* < 0.001; Fig. 1A). Immunohistochemical analysis of paraffin‐embedded sections from 145 pairs of lung adenocarcinomas and adjacent nontumor tissues revealed that SOX6 was frequently expressed in nontumor tissues (sections with overall scores ≥ 4, 132/145, 91.3%). In contrast, the levels of SOX6 were lower in lung adenocarcinomas (sections with overall scores ≥ 4, 57/145, 39%). The difference in SOX levels was statistically significant (*P* < 0.05). Western blot results further confirmed that the expression of SOX6 protein was down‐regulated in lung adenocarcinoma tissues compared with adjacent nontumor tissues (Fig. 1B,C).

### Low SOX6 expression is associated with poor prognosis of patients with lung adenocarcinoma

To assess the relationship between the expression of SOX6 and the clinicopathological features and prognosis of patients with lung adenocarcinoma, we compared SOX6 expression in the 145 lung adenocarcinomas with clinicopathological characteristics. We found that low SOX6 expression significantly correlated with poor differentiation (*P* < 0.001) and lymph node metastasis (*P* < 0.05; Table 1). Univariate analysis revealed that low SOX6 expression (*P* < 0.001), poor differentiation (*P* < 0.001), lymph node metastasis (*P* < 0.001) and late disease stage (*P* < 0.001) were significant prognostic factors (Table 2). Multivariate analysis showed that low SOX6 expression (*P* < 0.001), poor differentiation (*P* < 0.01) and late disease stage (*P* < 0.001) were independent prognostic factors (Table 3). These results suggest that patients with low Sox6 expression are often accompanied by poor differentiation and lymph node metastasis, and their prognosis is poor.

**Table 1:** Analysis of the relationship between clinicopathological characteristics and SOX6 expression. SOX6 expression was detected by immunohistochemistry.

Clinicopathological features	Cases, *n*	SOX6 expression, *n* (%)		χ^2^	*P*‐value
		Down‐regulation^a^	Normal^b^		
Tumor cell differentiation					
Well	52	19 (36.6)	33 (63.4)	21.5606	0.000*
Moderate	50	34 (68.0)	16 (32.0)		
Poor	43	35 (81.3)	8 (18.7)		
Sex					
Male	71	44 (62.0)	27 (38.0)	0.2665	0.606
Female	74	44 (60.0)	30 (40.0)		
Age, years					
≤60	77	46 (59.7)	31 (40.3)	0.062	0.803
>60	68	42 (61.8)	26 (38.2)		
Lymph node metastasis					
N0	93	50 (53.7)	43 (46.3)	5.2146	0.022*
N1	52	38 (73.1)	14 (26.9)		
TNM stage					
I	88	48 (54.5)	40 (45.5)	4.2988	0.117
II	35	23 (65.7)	12 (34.3)		
III	22	17 (77.3)	5 (22.7)		

^a^ Down‐regulation: sections with overall scores of 0–3

^b^ Normal: sections with overall scores ≥4

* *P* < 0.05.

**Table 2:** Univariate analysis of factors related to disease‐specific survival.

Variables	Hazard ratio	95% CI	*P*‐value
SOX6	0.377	0.258–0.551	0.000*
Sex	1.077	0.761–1.523	0.676
Age	1.009	0.986–1.032	0.464
Differentiation	1.848	1.467–2.327	0.000*
Lymph nodes metastasis	2.205	1.532–3.179	0.000*
Stage	1.898	1.493–2.412	0.000*

* *P* < 0.05.

**Table 3:** Multivariate analysis of factors related to disease‐specific survival.

Variables	Hazard ratio	95% CI	*P*‐value
SOX6	0.463	0.310–0.702	0.000*
Sex	1.319	0.915–1.901	0.138
Age	1.028	1.003–1.054	0.029*
Differentiation	1.493	1.139–1.956	0.004*
Lymph nodes metastasis	1.487	0.907–2.437	0.116
Stage	2.836	1.580–5.090	0.000*

* *P* < 0.05.

**Figure 2:**
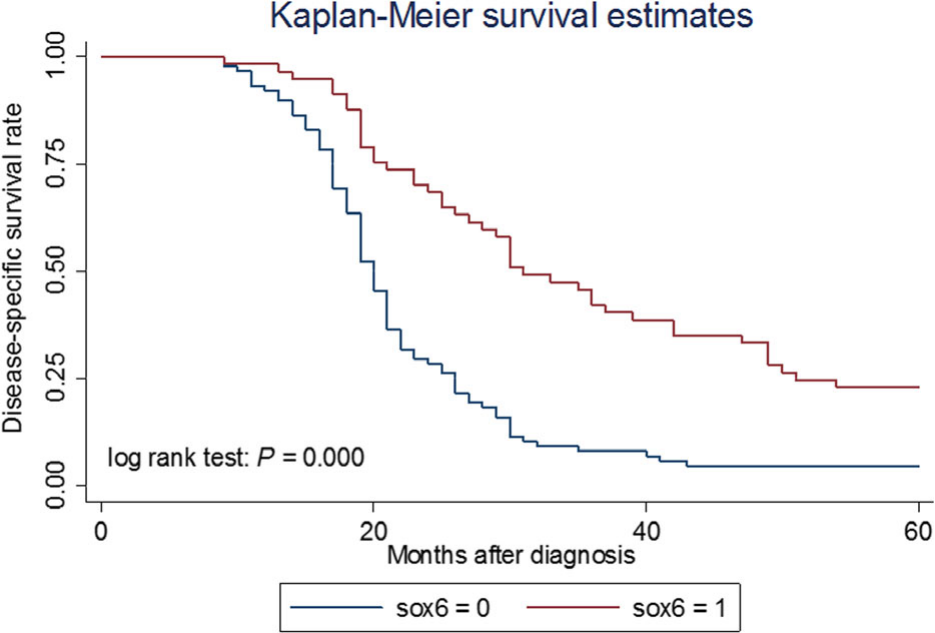
Kaplan–Meier analysis of the relationship between survival and SOX6 expression in patients with lung adenocarcinoma. SOX6 = 1 (staining score ≥ 4), patients with normal levels of SOX6 expression (*n* = 57; median survival, 36.3 months; brown curve); SOX6 = 0 (staining scores = 0–3), patients with down‐regulation of SOX6 expression (*n* = 88; median survival, 22.6 months; blue curve); log rank test, *P* < 0.001.

The log rank test showed that disease‐related survival (date of diagnosis to the date of cancer‐related death or last follow‐up) of lung adenocarcinoma patients with low SOX6 expression was significantly shorter compared with that of patients with normal levels of SOX6 (median survival, 22.6 versus 36.3 months, respectively; *P* < 0.001; Fig. 2).

### SOX6 inhibits the development and metastasis of lung adenocarcinoma

**Figure 3:**
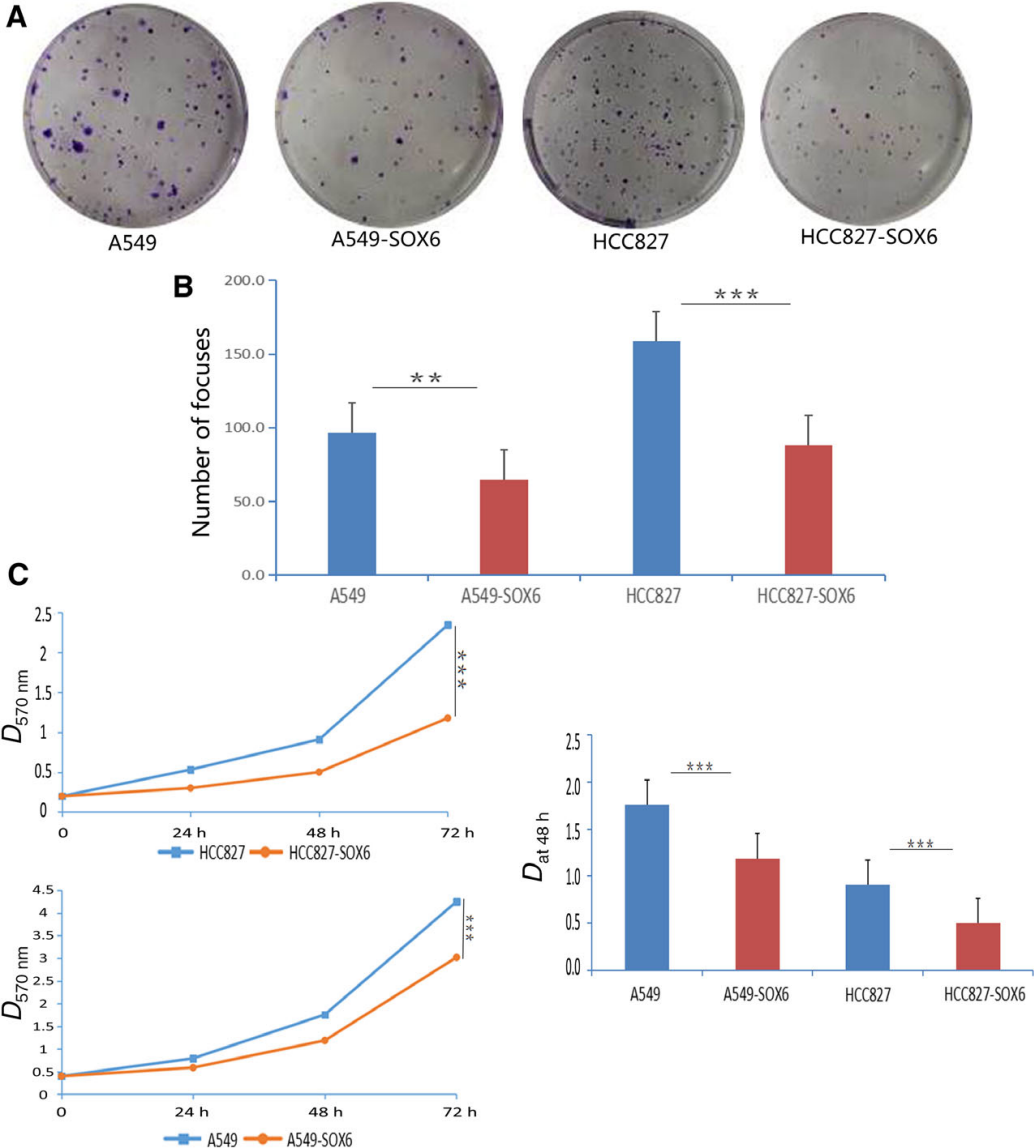
SOX6 inhibits the proliferation of lung adenocarcinoma cells. (A) Example of inhibition of colony formation in a monolayer culture by SOX6. (B) Number of colonies versus controls. **P < 0.05, ***P < 0.01. (C) Growth curves of SOX6‐HCC827 and Vec‐HCC827 cells and SOX6‐A549 and Vec‐A549 cells (left). Histogram of the number of cells in cell cultures after 48 h (right). *n* = 3. Student’s *t*‐test was used. Error bars represent SD. ***P < 0.01.

We next evaluated the effect of SOX6 on tumor formation and cell proliferation in cell proliferation and colony formation assays using cell lines stably expressing SOX6 (SOX6‐A549 and SOX6‐HCC827). Both SOX6‐A549 and SOX6‐HCC827 cells showed significantly reduced colony formation activity compared with control cells transfected with the empty vector (Vec‐A549 and Vec‐HCC827; SOX6‐A549 cells, *P* < 0.05; SOX6‐HCC827 cells, *P* < 0.01; Fig. 3A,B). MTT assays revealed that proliferation of SOX6‐A549 and SOX6‐HCC827 cells was significantly inhibited compared with the respective empty vector‐transfected controls (*P* < 0.01; Fig. 3C).

**Figure 4:**
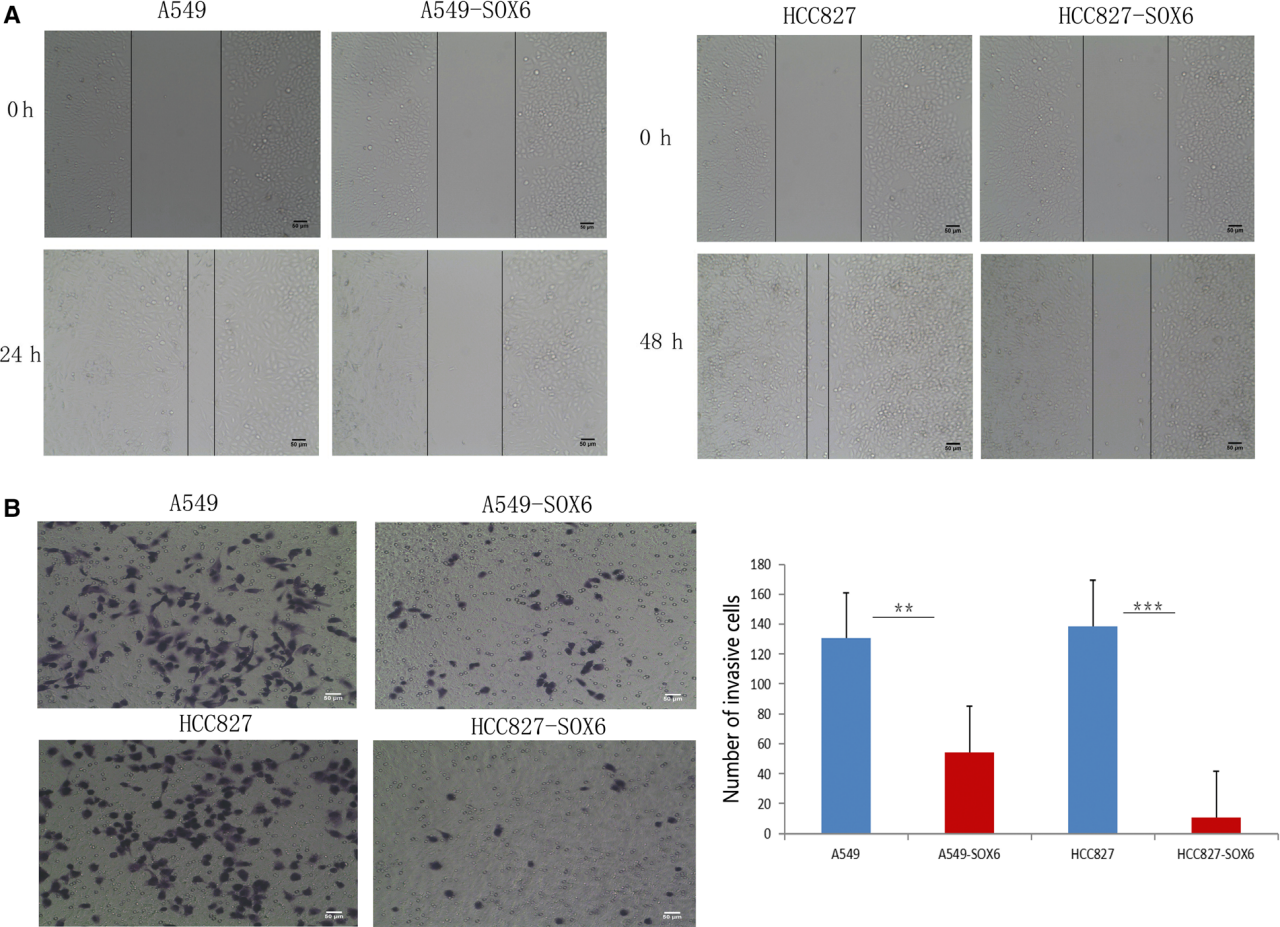
SOX6 inhibits the invasiveness of lung adenocarcinoma cells. (A) The effect of SOX6 on the migration of lung adenocarcinoma cells detected using a wound healing assay. Cells stably expressing SOX6 (SOX6‐A549 and SOX6‐HCC827) migrated slower than their respective control cells. (B) Representative images of SOX6‐A549 and SOX6‐HCC827 cells that invaded through Matrigel (left). Numbers of invading tumor cells (right). *n* = 3. Student’s *t*‐test was used. Error bars represent SD. **P < 0.05, ***P < 0.01. Scale bars: 50 μm.

We further examined the effect of SOX6 expression on the invasiveness of lung adenocarcinoma cells using wound healing and Transwell assays. The migration of SOX6‐A549 and SOX6‐HCC827 cells was significantly reduced compared with the respective controls (Fig. 4A). Similarly, the invasiveness of the stable SOX6‐expressing clones (SOX6‐A549 and SOX6‐HCC827) was reduced compared with the empty vector‐transfected controls (SOX6‐A549 cells, *P* < 0.05; SOX6‐HCC827 cells, *P* < 0.01; Fig. 4B).

### The mechanism of the inhibitory effect of SOX6 on the cell cycle of lung adenocarcinoma cells

**Figure 5:**
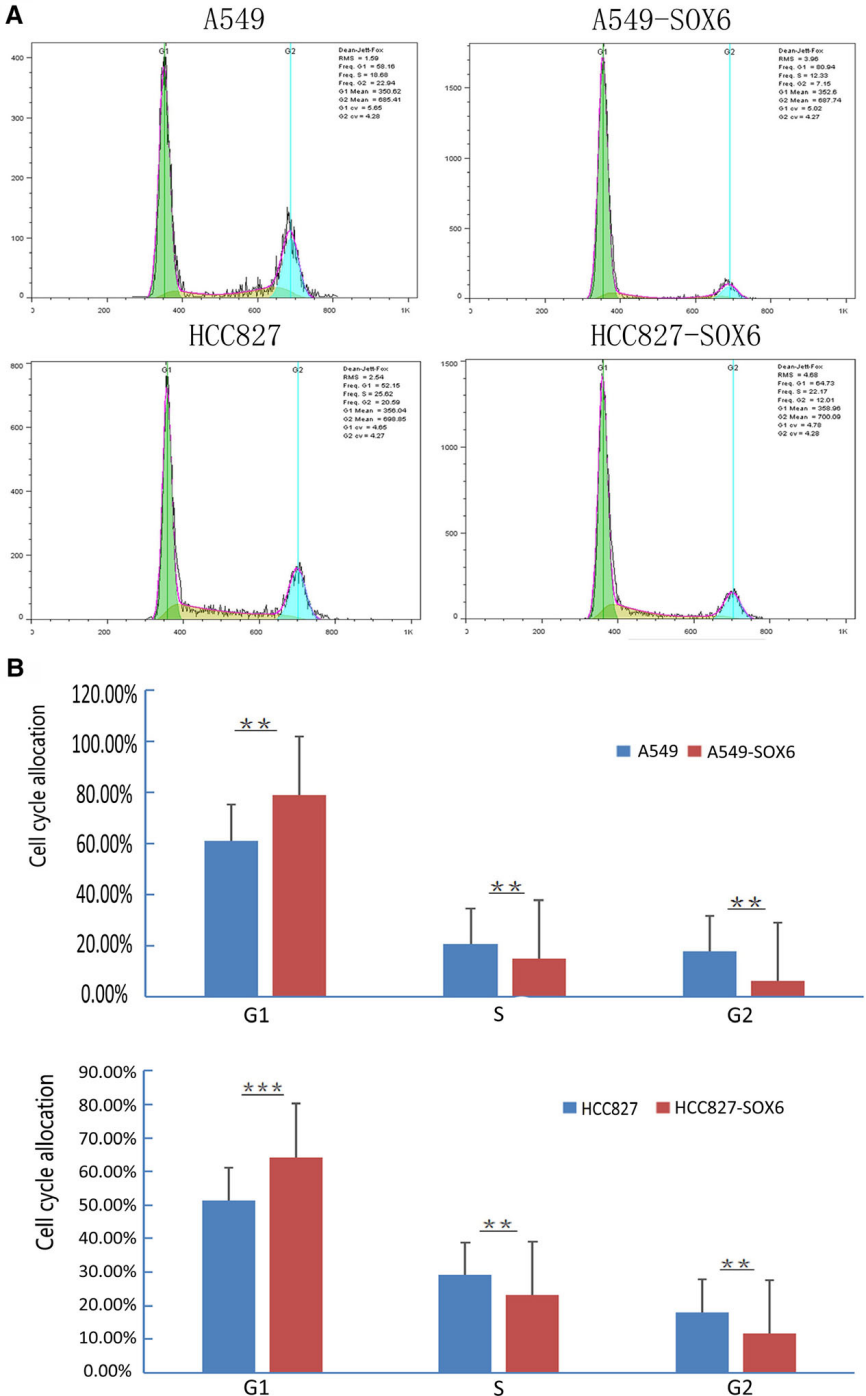
SOX6 inhibits the G1/S transition. (A) Flow cytometry was performed to assess the cell cycle. The percentage of cells in S phase was lower in SOX6‐A549 and SOX6‐HCC827 cells compared with those of the respective control cells. (B) Histogram of cell ratio at each phase in the stable SOX6‐expressing clones (SOX6‐A549 and SOX6‐HCC827) compared with the untransfected controls (Vec‐A549 and Vec‐HCC827). *n* = 3. Student’s *t*‐test was used. Error bars represent SD. **P < 0.05, ***P < 0.01.

**Figure 6:**
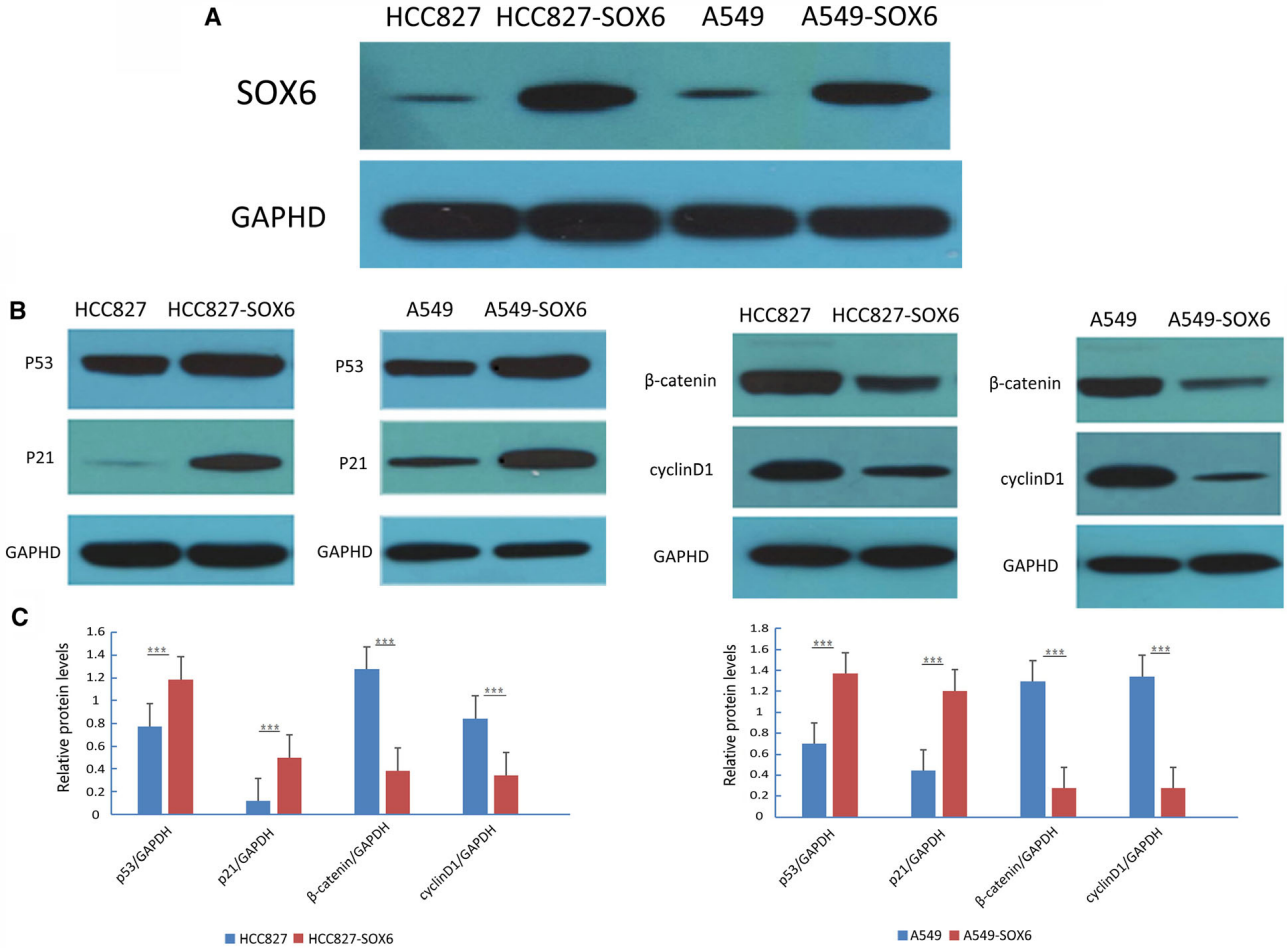
Regulation of Sox6 on p53, p21^CIPI^, β‐catenin and Cyclin D1. (A) Western blot was used to detect the overexpression of SOX6 in stably transfected A549 and HCC827 cell lines. Empty vector‐transfected cells (Vec‐A549/Vec‐HCC827) were used as controls. (B) Western blot analysis of the expression of p53, p21^CIPI^, β‐catenin and cyclin D1 in SOX6‐A549 and SOX6‐HCC827 cells. GAPDH served as a loading control. (C) Histogram of protein expression in the stable SOX6‐expressing clones (SOX6‐A549 and SOX6‐HCC827) compared with the untransfected controls (Vec‐A549 and Vec‐HCC827). *n* = 3. Student’s *t*‐test was used. Error bars represent SD. ***P < 0.001.

To identify the mechanism through which SOX6 inhibits tumor growth, we next analyzed the cell cycle. Flow cytometry revealed that the numbers of SOX6‐A549 and SOX6‐HCC827 cells in the G0/G1 phase were significantly increased compared with controls, with a significant decrease in S phase cells, indicating that SOX6 inhibited the G1/S transition (*P* < 0.05; Fig. 5A,B). Overexpression of SOX6 in SOX6‐transfected lung cancer cells (SOX6‐A549 and SOX6‐HCC827) was confirmed by western blot analysis (Fig. 6A). We next performed western blotting to investigate the effects of SOX6 on the levels of cell cycle regulatory proteins (p53, p21^CIPI^, cyclin D1 and β‐catenin). The levels of p53 and p21^CIPI^ were significantly increased in SOX6 stable cells compared with controls, whereas the levels of cyclin D1 and β‐catenin were significantly decreased (*P* < 0.001; Fig. 6B,C).

## Discussion

The gene‐encoding transcription factor SOX6, which was first isolated from mouse testes 24, is located in homologous chromosome region 11p15.3‐15.2 and expressed in various tissues. Recent studies have demonstrated that SOX6 not only plays an important role in chondrocyte and skeletal muscle differentiation, but also regulates erythropoiesis, myocardial cell proliferation and insulin secretion 25. It has been reported that children with SOX6 mutations experience development of global developmental delay, progressive relapsing–remitting parkinsonism and spinal syrinx 25. Some studies have shown that the abnormal expression of Sox6 is related to the occurrence and development of cancer. For example, down‐regulation of SOX6 expression in pancreatic cancer, esophageal cancer and ovarian cancer can inhibit the proliferation, migration and invasion of cancer cells, thus inhibiting the invasion and metastasis of cancer 14, 15, 26.

The abnormal expression of SOX6 is also associated with the prognosis of some tumors. SOX6 acts as a tumor suppressor in esophageal cancer and is an independent prognostic factor of esophageal cancer 9. The expression level of SOX6 in hepatocellular carcinoma is negatively associated with tumor stage, and patients with down‐regulated expression of SOX6 show poor prognosis 8. At present, whether the expression of SOX6 is related to the prognosis of lung cancer is not clear.

No studies have explored the role of SOX6 in lung cancer; thus, our current research provides some preliminary data in this area and suggests a new direction for further exploration of new targets in lung adenocarcinoma.

We showed that the expression of SOX6 in lung adenocarcinoma tissues was down‐regulated at both the protein and the mRNA level. These results suggest that SOX6 may function as a tumor suppressor in lung adenocarcinoma, and the down‐regulation of SOX6 expression may be related to the occurrence and development of lung adenocarcinoma.

To investigate the clinical significance of down‐regulation of SOX6 expression in lung adenocarcinoma, we retrospectively analyzed the clinical data of 145 patients with lung adenocarcinoma. The results showed that the expression of SOX6 was closely related to the degree of differentiation and lymph node metastasis. Patients with poorly differentiated or lymph node metastasis often also showed down‐regulation of SOX6 expression. Univariate and multivariate analyses suggest that SOX6 expression was related to disease‐related survival and might be an independent prognostic factor. Survival analysis showed that patients with down‐regulated SOX6 expression had significantly shortened disease‐related survival and poor prognosis compared with patients with normal expression of Sox6. These results suggest that SOX6 expression may serve as a potential prognostic marker.

In this study, we found that SOX6 was down‐regulated in patients with lung adenocarcinoma, and *in vitro* cell experiments indicated that SOX6 inhibits the proliferation, migration and invasion of lung adenocarcinoma cells; thus, SOX6 may possibly function as a tumor suppressor. These results are consistent with those reported in the literature regarding the role of SOX6 in other tumors. For example, in prostate cancer, miRNA‐671 promotes tumor proliferation by inhibiting the expression of SOX6 14. Furthermore, miRNA‐96 and miRNA‐155 decrease proliferation, migration and invasion of hepatocellular carcinoma cells by inhibiting the expression of SOX6 13, 27. These studies support the inhibitory activity of SOX6 in tumors.

The oncogenesis of lung cancer involves the activities of numerous signaling pathways, particularly the Wnt/β‐catenin signaling pathways 28, 29. We demonstrated that SOX6 reduced the expression of β‐catenin in a lung adenocarcinoma cell line, suggesting that the tumor suppressor function of SOX6 may be associated with this signaling pathway. Wnt/β‐catenin signaling pathways mediate diverse processes, including cell proliferation, migration, differentiation and death 30. The core feature of these mechanisms is the regulation of the stability of β‐catenin. β‐Catenin is a multifunctional protein that interacts with E‐cadherin at cell junctions and participates in the formation of adhesions, whereas free β‐catenin enters the nucleus to form a complex with lymphoid‐enhancing factor/T cell factor 31, 32. This complex acts as a transcriptional regulator for many genes and activates downstream target genes myelocytomatosis oncogene and cyclin D1 to promote tumor cell differentiation and proliferation 33, 34.

Cyclin D1 is G1/S‐specific cyclin 35. p21^CIPI^ is a cyclin‐dependent kinase inhibitor that negatively regulates the cell cycle and inhibits the growth of tumor cells 36. The gene encoding p21^CIPI^ is also the main downstream target gene of p53. p53 represses G1 cell cycle progression by up‐regulating p21^CIPI^, and the G1/S transition is the key to the cell cycle 37. Recent studies have shown that SOX6 up‐regulates the expression of p21^CIPI^ protein in a high‐mobility group domain‐dependent manner through the p14ARF‐HDM2‐p53 axis to play a role in tumor inhibition 38. To clarify the mechanism of SOX6 in lung adenocarcinoma, we carried out cell cycle and immunoblotting assays. We found that lung adenocarcinoma cells stably transfected with SOX6 showed increased numbers of cells in G1 phase, indicating that SOX6 negatively regulates G1 phase transition in lung adenocarcinoma cells. These data suggest that SOX6 may function as a tumor suppressor by regulating the cell cycle in lung adenocarcinoma. We also found that p53 and p21^CIPI^ levels were significantly increased in lung adenocarcinoma cells transfected with SOX6, whereas the expressions of β‐catenin and cyclin D1 were significantly decreased. This result was consistent with the role of SOX6 in esophageal squamous cell carcinoma 9. These findings suggest that the inhibitory effect of SOX6 in lung adenocarcinoma may be related to the inhibition of G1 transition through up‐regulation of p53 and p21^CIPI^ expression and down‐regulation of β‐catenin and cyclin D1 expression. Animal experiments will be required to confirm whether SOX6 acts as a tumor suppressor.

## Conclusions

Our study suggests that SOX6 may be a tumor suppressor in lung adenocarcinoma, and our results show that the prognosis of patients with lung adenocarcinoma with low SOX6 expression is poor. These findings suggest that SOX6 may be a potential new therapeutic target and molecular predictor of lung adenocarcinoma.

## Conflict of interest

The authors declare no conflict of interest.

## Author contributions

LL was responsible for literature review, experiment design, experiment execution, data collection and compilation, as well as manuscript writing. LL, MZ and JZ were responsible for specimen collection and experiment execution. FL and LQ were responsible for statistical analysis. LL and SZ were responsible for collecting clinical case data. LL and YB were responsible for literature review. YY was responsible for overall experimental design, feasibility analysis, experimental process supervision, and manuscript review and revision.

## Supporting information


**Table S1.** Clinical features of 30 patients with primary lung adenocarcinoma.
**Table S2.** Clinicopathological features and SOX6 expression in 145 informative patients with lung adenocarcinoma.
