# Corrigendum to: SOX6 suppresses the development of lung adenocarcinoma by regulating expression of p53, p21^CIPI^, cyclin D1 and β‐catenin

**DOI:** 10.1002/2211-5463.12797

**Published:** 2020-02-12

In the original article, there were errors in the calculation of standard deviation and *P* values shown in Figure 5 and in the chi square test for gender in Table 1. Corrected versions of Figure 5 and Table 1 are provided here. This does not affect the conclusions of the paper.

**Figure 5:**
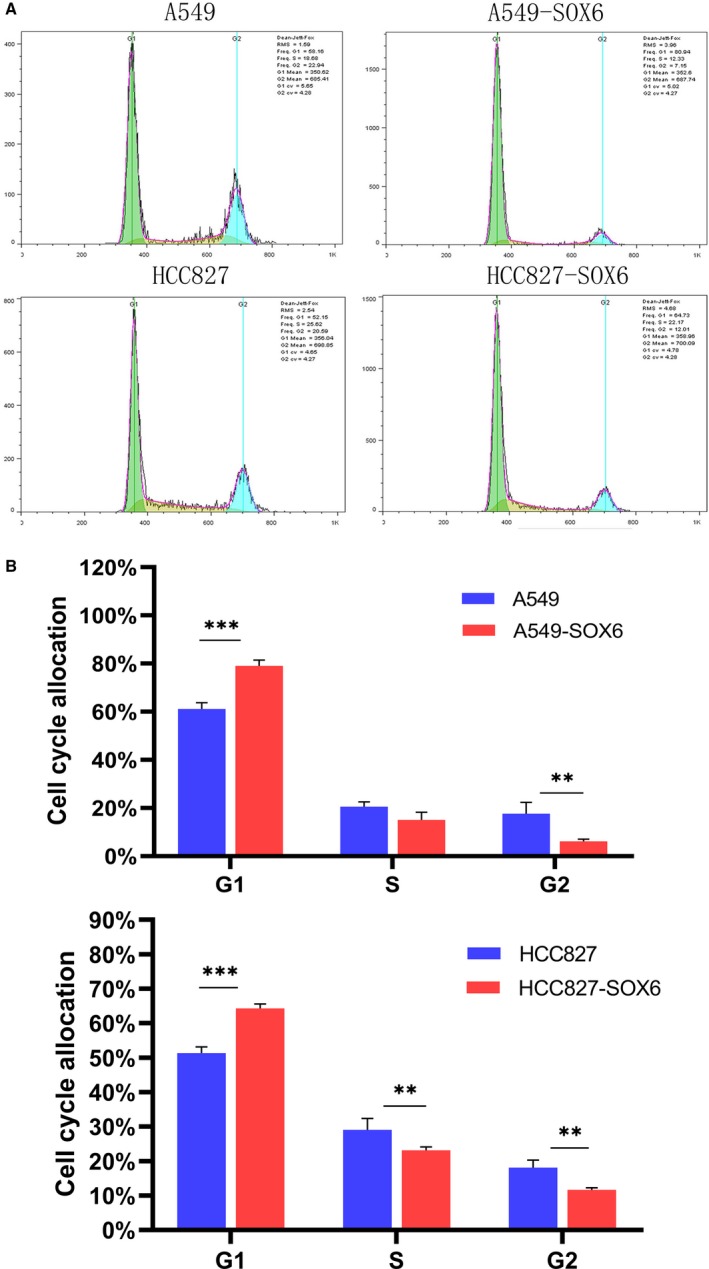
SOX6 inhibits the G1/S transition. (A) Flow cytometry was performed to assess the cell cycle. The percentage of cells in G1 phase was higher in SOX6‐A549 and SOX6‐HCC827 cells compared with those of the respective control cells. (B) Histogram of cell ratio at each phase in the stable SOX6‐expressing clones (SOX6‐A549 and SOX6‐HCC827) compared with the controls (Vec‐A549 and Vec‐HCC827). *n* = 3. Student’s *t*‐test was used. Error bars represent SD. **P < 0.05, ***P < 0.01.

**Table 1:** Analysis of the relationship between patients' clinicopathological characteristics and SOX6 expression.

Clinicopathological features	Case, *n*	sox6 expression, *n *(%)		*χ* ^2^	*P*‐value
		Down‐regulation^a^	Normal^b^		
Tumor cell differentiation					
Well	52	19 (36.6)	33 (63.4)	21.5606	0.000*
Moderate	50	34 (68.0)	16 (32.0)		
Poor	43	35 (81.3)	8 (18.7)		
Gender					
Male	71	44 (62.0)	27 (38.0)	0.096	0.757
Female	74	44 (60.0)	30 (40.0)		
Age, y					
≤ 60	77	46 (59.7)	31 (40.3)	0.062	0.803
> 60	68	42 (61.8)	26 (38.2)		
Lymph nodes metastasis (N)					
N0	93	50 (53.7)	43 (46.3)	5.2146	0.022*
N1	52	38 (73.1)	14 (26.9)		
TNM stage					
I	88	48 (54.5)	40 (45.5)	4.2988	0.117
II	35	23 (65.7)	12 (34.3)		
III	22	17 (77.3)	5 (22.7)		

a Down‐regulation: sections with overall scores of 0–3; ^b^Normal: sections with overall scores ≥ 4; *P < 0.05.

## Reference

Lv L, Zhou M, Zhang J, Liu F, Qi L, Zhang S, Bi Y and Yu Y (2020) SOX6 suppresses the development of lung adenocarcinoma by regulating expression of p53, p21^CIPI^, cyclin D1 and β‐catenin. *Febs Open Bio* **10**, 135–146.

