# Supplementary material for: SOX6 suppresses the development of lung adenocarcinoma by regulating expression of p53, p21CIPI, cyclin D1 and β‐catenin
Source: FEBS Open Bio. 2019 Dec 12;10(1):135–46. doi: 10.1002/2211-5463.12762 (PMC6943225; doi:10.1002/2211-5463.12762)
Supplement: Supplementary file 1 — Table S1. Clinical features of 30 patients with primary lung adenocarcinoma. Table S2. Clinicopathological features and SOX6 expression in 145 informative patients with lung adenocarcinoma. [file FEB4-10-135-s001.docx]

| Supplementary Table 1. Clinical features of 30 patients with primary lung adenocarcinoma. | |
| --- | --- |
| Patient characteristie | n=30 |
| Age |  |
| <=60 | 18 |
| >60 | 12 |
| Gender |  |
| Female | 20 |
| Male | 10 |
| pT |  |
| T1 | 3 |
| T2 | 14 |
| T3 | 12 |
| T4 | 1 |
| pN |  |
| N0 | 19 |
| N1 | 11 |

| Supplementary Table 2. Clinicopathological features and SOX6 expression in 145 informative patients with lung adenocarcinoma. | | | | | | | | |
| --- | --- | --- | --- | --- | --- | --- | --- | --- |
| NO | SOX6^a^ | Gender^b^ | Age | Survival | Censor^c^ | Differ^d^ | LN.Met^e^ | Stage |
| 1 | 1 | 0 | 67 | 42 | 0 | 1 | 0 | I |
| 2 | 1 | 1 | 65 | 60 | 1 | 1 | 0 | I |
| 3 | 1 | 0 | 61 | 30 | 0 | 1 | 0 | I |
| 4 | 1 | 0 | 73 | 35 | 0 | 1 | 0 | I |
| 5 | 1 | 1 | 53 | 60 | 1 | 1 | 0 | I |
| 6 | 1 | 0 | 73 | 23 | 0 | 1 | 0 | I |
| 7 | 1 | 0 | 64 | 19 | 0 | 1 | 0 | I |
| 8 | 1 | 1 | 48 | 60 | 1 | 1 | 0 | I |
| 9 | 1 | 0 | 68 | 30 | 0 | 2 | 0 | I |
| 10 | 1 | 0 | 55 | 37 | 0 | 1 | 0 | I |
| 11 | 1 | 1 | 65 | 17 | 0 | 1 | 0 | I |
| 12 | 1 | 0 | 58 | 60 | 1 | 1 | 0 | I |
| 13 | 1 | 1 | 64 | 27 | 0 | 1 | 0 | I |
| 14 | 1 | 0 | 55 | 60 | 1 | 1 | 0 | I |
| 15 | 1 | 0 | 62 | 29 | 0 | 1 | 0 | I |
| 16 | 1 | 1 | 53 | 60 | 1 | 2 | 0 | I |
| 17 | 1 | 1 | 68 | 20 | 0 | 1 | 0 | I |
| 18 | 1 | 0 | 55 | 47 | 0 | 2 | 0 | I |
| 19 | 1 | 0 | 63 | 24 | 0 | 2 | 0 | I |
| 20 | 1 | 1 | 61 | 54 | 0 | 2 | 0 | I |
| 21 | 1 | 1 | 60 | 50 | 0 | 1 | 0 | I |
| 22 | 1 | 1 | 51 | 33 | 0 | 1 | 0 | I |
| 23 | 1 | 1 | 56 | 18 | 0 | 2 | 0 | I |
| 24 | 1 | 0 | 61 | 49 | 0 | 1 | 0 | I |
| 25 | 1 | 1 | 59 | 30 | 0 | 1 | 0 | I |
| 26 | 1 | 0 | 62 | 60 | 1 | 1 | 0 | I |
| 27 | 1 | 0 | 53 | 14 | 0 | 2 | 0 | I |
| 28 | 1 | 0 | 49 | 60 | 1 | 1 | 0 | I |
| 29 | 1 | 1 | 67 | 26 | 0 | 1 | 0 | I |
| 30 | 1 | 1 | 66 | 60 | 1 | 1 | 0 | I |
| 31 | 1 | 0 | 62 | 19 | 0 | 1 | 0 | I |
| 32 | 1 | 1 | 61 | 51 | 0 | 1 | 0 | I |
| 33 | 1 | 0 | 63 | 36 | 0 | 1 | 0 | I |
| 34 | 1 | 1 | 60 | 30 | 0 | 2 | 0 | I |
| 35 | 1 | 1 | 69 | 42 | 0 | 1 | 0 | I |
| 36 | 1 | 0 | 70 | 21 | 0 | 1 | 0 | I |
| 37 | 1 | 0 | 58 | 49 | 0 | 1 | 0 | I |
| 38 | 1 | 1 | 61 | 17 | 0 | 1 | 0 | I |
| 39 | 1 | 0 | 50 | 49 | 0 | 2 | 0 | I |
| 40 | 1 | 1 | 56 | 39 | 0 | 1 | 0 | I |
| 41 | 0 | 0 | 70 | 23 | 0 | 1 | 0 | I |
| 42 | 0 | 0 | 74 | 22 | 0 | 1 | 0 | I |
| 43 | 0 | 1 | 69 | 31 | 0 | 1 | 0 | I |
| 44 | 0 | 1 | 65 | 26 | 0 | 1 | 0 | I |
| 45 | 0 | 1 | 64 | 26 | 0 | 2 | 0 | I |
| 46 | 0 | 0 | 68 | 19 | 0 | 1 | 0 | I |
| 47 | 0 | 1 | 69 | 19 | 0 | 2 | 0 | I |
| 48 | 0 | 1 | 75 | 22 | 0 | 1 | 0 | I |
| 49 | 0 | 0 | 70 | 30 | 0 | 1 | 0 | I |
| 50 | 0 | 0 | 67 | 35 | 0 | 1 | 0 | I |
| 51 | 0 | 0 | 63 | 29 | 0 | 2 | 0 | I |
| 52 | 0 | 0 | 68 | 22 | 0 | 1 | 0 | I |
| 53 | 0 | 0 | 64 | 18 | 0 | 2 | 0 | I |
| 54 | 0 | 1 | 48 | 41 | 0 | 2 | 0 | I |
| 55 | 0 | 0 | 74 | 23 | 0 | 3 | 0 | I |
| 56 | 0 | 1 | 66 | 19 | 0 | 1 | 0 | I |
| 57 | 0 | 1 | 67 | 20 | 0 | 2 | 0 | I |
| 58 | 0 | 1 | 57 | 17 | 0 | 2 | 0 | I |
| 59 | 0 | 0 | 69 | 20 | 0 | 1 | 0 | I |
| 60 | 0 | 0 | 52 | 16 | 0 | 3 | 0 | I |
| 61 | 0 | 1 | 61 | 27 | 0 | 2 | 0 | I |
| 62 | 0 | 1 | 61 | 32 | 0 | 2 | 0 | I |
| 63 | 0 | 0 | 73 | 20 | 0 | 2 | 0 | I |
| 64 | 0 | 1 | 48 | 43 | 0 | 2 | 0 | I |
| 65 | 0 | 1 | 53 | 21 | 0 | 3 | 0 | I |
| 66 | 0 | 0 | 62 | 60 | 1 | 1 | 0 | I |
| 67 | 0 | 1 | 61 | 19 | 0 | 2 | 0 | I |
| 68 | 0 | 0 | 55 | 25 | 0 | 2 | 0 | I |
| 69 | 0 | 0 | 67 | 60 | 1 | 3 | 0 | I |
| 70 | 0 | 0 | 53 | 30 | 0 | 3 | 0 | I |
| 71 | 0 | 0 | 52 | 17 | 0 | 3 | 0 | I |
| 72 | 0 | 1 | 62 | 19 | 0 | 3 | 0 | I |
| 73 | 0 | 0 | 61 | 15 | 0 | 3 | 0 | I |
| 74 | 0 | 1 | 84 | 26 | 0 | 1 | 0 | I |
| 75 | 0 | 0 | 48 | 16 | 0 | 3 | 0 | I |
| 76 | 0 | 1 | 69 | 21 | 0 | 2 | 0 | I |
| 77 | 0 | 1 | 46 | 18 | 0 | 3 | 0 | I |
| 78 | 0 | 0 | 59 | 25 | 0 | 2 | 0 | I |
| 79 | 0 | 0 | 65 | 30 | 0 | 1 | 0 | I |
| 80 | 0 | 1 | 58 | 27 | 0 | 3 | 0 | I |
| 81 | 0 | 1 | 55 | 60 | 1 | 2 | 0 | I |
| 82 | 0 | 1 | 62 | 40 | 0 | 1 | 0 | I |
| 83 | 0 | 0 | 64 | 17 | 0 | 2 | 0 | I |
| 84 | 0 | 0 | 44 | 11 | 0 | 3 | 0 | I |
| 85 | 0 | 1 | 59 | 28 | 0 | 3 | 0 | I |
| 86 | 0 | 1 | 67 | 20 | 0 | 3 | 0 | I |
| 87 | 0 | 1 | 63 | 24 | 0 | 3 | 0 | I |
| 88 | 0 | 1 | 63 | 30 | 0 | 2 | 0 | I |
| 89 | 1 | 1 | 59 | 23 | 0 | 1 | 0 | II |
| 90 | 1 | 1 | 59 | 20 | 0 | 2 | 1 | II |
| 91 | 1 | 0 | 44 | 60 | 1 | 1 | 0 | II |
| 92 | 1 | 1 | 49 | 19 | 0 | 2 | 1 | II |
| 93 | 1 | 1 | 54 | 60 | 1 | 2 | 1 | II |
| 94 | 1 | 0 | 52 | 31 | 0 | 2 | 1 | II |
| 95 | 1 | 1 | 62 | 36 | 0 | 2 | 1 | II |
| 96 | 1 | 0 | 52 | 28 | 0 | 3 | 1 | II |
| 97 | 1 | 0 | 64 | 19 | 0 | 3 | 1 | II |
| 98 | 1 | 0 | 59 | 60 | 1 | 2 | 1 | II |
| 99 | 1 | 1 | 60 | 18 | 0 | 3 | 1 | II |
| 100 | 1 | 0 | 46 | 19 | 0 | 3 | 1 | II |
| 101 | 0 | 1 | 60 | 21 | 0 | 2 | 1 | II |
| 102 | 0 | 1 | 61 | 16 | 0 | 2 | 1 | II |
| 103 | 0 | 1 | 72 | 21 | 0 | 1 | 0 | II |
| 104 | 0 | 0 | 53 | 21 | 0 | 2 | 1 | II |
| 105 | 0 | 0 | 52 | 18 | 0 | 1 | 0 | II |
| 106 | 0 | 1 | 61 | 18 | 0 | 2 | 1 | II |
| 107 | 0 | 0 | 49 | 19 | 0 | 3 | 1 | II |
| 108 | 0 | 0 | 55 | 14 | 0 | 3 | 1 | II |
| 109 | 0 | 0 | 52 | 60 | 1 | 2 | 1 | II |
| 110 | 0 | 1 | 51 | 21 | 0 | 3 | 1 | II |
| 111 | 0 | 1 | 49 | 20 | 0 | 3 | 1 | II |
| 112 | 0 | 0 | 62 | 17 | 0 | 2 | 1 | II |
| 113 | 0 | 1 | 49 | 17 | 0 | 2 | 1 | II |
| 114 | 0 | 1 | 57 | 22 | 0 | 2 | 1 | II |
| 115 | 0 | 0 | 58 | 19 | 0 | 3 | 1 | II |
| 116 | 0 | 1 | 51 | 19 | 0 | 1 | 0 | II |
| 117 | 0 | 0 | 43 | 21 | 0 | 3 | 1 | II |
| 118 | 0 | 1 | 50 | 16 | 0 | 3 | 1 | II |
| 119 | 0 | 0 | 74 | 21 | 0 | 3 | 1 | II |
| 120 | 0 | 1 | 57 | 17 | 0 | 3 | 1 | II |
| 121 | 0 | 0 | 56 | 29 | 0 | 3 | 1 | II |
| 122 | 0 | 1 | 45 | 19 | 0 | 3 | 1 | II |
| 123 | 0 | 0 | 47 | 18 | 0 | 3 | 1 | II |
| 124 | 1 | 1 | 56 | 25 | 0 | 3 | 1 | III |
| 125 | 1 | 1 | 35 | 9 | 0 | 3 | 1 | III |
| 126 | 1 | 0 | 46 | 60 | 1 | 2 | 1 | III |
| 127 | 1 | 0 | 53 | 13 | 0 | 3 | 1 | III |
| 128 | 1 | 0 | 54 | 25 | 0 | 3 | 1 | III |
| 129 | 0 | 0 | 59 | 17 | 0 | 2 | 1 | III |
| 130 | 0 | 0 | 58 | 19 | 0 | 2 | 1 | III |
| 131 | 0 | 0 | 74 | 26 | 0 | 1 | 1 | III |
| 132 | 0 | 1 | 60 | 14 | 0 | 3 | 1 | III |
| 133 | 0 | 0 | 57 | 11 | 0 | 3 | 1 | III |
| 134 | 0 | 1 | 62 | 9 | 0 | 3 | 1 | III |
| 135 | 0 | 0 | 52 | 15 | 0 | 2 | 1 | III |
| 136 | 0 | 1 | 53 | 14 | 0 | 2 | 1 | III |
| 137 | 0 | 0 | 54 | 9 | 0 | 3 | 1 | III |
| 138 | 0 | 0 | 55 | 20 | 0 | 3 | 1 | III |
| 139 | 0 | 0 | 72 | 13 | 0 | 2 | 1 | III |
| 140 | 0 | 1 | 59 | 10 | 0 | 3 | 1 | III |
| 141 | 0 | 1 | 65 | 13 | 0 | 2 | 1 | III |
| 142 | 0 | 0 | 77 | 11 | 0 | 2 | 1 | III |
| 143 | 0 | 0 | 54 | 17 | 0 | 3 | 1 | III |
| 144 | 0 | 1 | 57 | 12 | 0 | 2 | 1 | III |
| 145 | 0 | 1 | 57 | 15 | 0 | 3 | 1 | III |

^a^SOX6: 1= normal, 0= downregulated;

^b^Cender: 0=Female, 1=male;

^c^Different: 1= well differentiation, 2= moderate differentiation, 3= poor differentiation;

^d^LN.Met: 0= no LN metastasis, 1= with LN metastasis;

^e^Censor: 0=ded at the end of the follow-up,

1=still alive at the end of the follow-up.
